# The electrocardiographic, hemodynamic, echocardiographic, and biochemical evaluation of treatment with edaravone on acute cardiac toxicity of aluminum phosphide

**DOI:** 10.3389/fphar.2022.1032941

**Published:** 2022-10-05

**Authors:** Nader Rahimi Kakavandi, Tayebeh Asadi, Mohammad Reza Hooshangi Shayesteh, Maryam Baeeri, Mahban Rahimifard, Amir Baghaei, Marzieh Noruzi, Mohammad Sharifzadeh, Mohammad Abdollahi

**Affiliations:** ^1^ Department of Toxicology & Pharmacology, Faculty of Pharmacy, Tehran University of Medical Sciences (TUMS), Tehran, Iran; ^2^ Health and Environment Research Center, Ilam University of Medical Sciences, Ilam, Iran; ^3^ Toxicology and Diseases Group, Pharmaceutical Sciences Research Center (PSRC), The Institute of Pharmaceutical Sciences (TIPS), Tehran University of Medical Sciences (TUMS), Tehran, Iran; ^4^ Department of Toxicology and Pharmacology, Faculty of Pharmacy, Alborz University of Medical Sciences, Karaj, Iran

**Keywords:** aluminum phosphide, phosphine, edaravone, cardiotoxicity, oxidative stress, apoptosis, mitochondrial toxicity

## Abstract

Aluminum phosphide (AlP) poisoning can be highly fatal due to its severe toxicity to the heart. Based on the evidence, edaravone (EDA) has protective effects on various pathological conditions of the heart. This research aimed to examine the potential protective effects of EDA on AlP-induced cardiotoxicity in rats. The rats were divided into six groups, including almond oil (control), normal saline, AlP (LD_50_), and AlP + EDA (20, 30, and 45 mg/kg). Thirty minutes following AlP poisoning, the electrocardiographic (ECG), blood pressure (BP), and heart rate (HR) parameters were examined for 180 min. The EDA was injected 60 min following the AlP poisoning intraperitoneally. Also, 24 h after poisoning, echocardiography was carried out to evaluate the ejection fraction (EF), stroke volume (SV), and cardiac output (CO). The biochemical and molecular parameters, such as the activities of the mitochondrial complexes, reactive oxygen species (ROS), apoptosis and necrosis, and troponin I and lactate levels, were also examined after 12 and 24 h in the heart tissue. According to the results, AlP-induced ECG abnormalities, decrease in blood pressure, heart rate, SV, EF%, and CO were significantly improved with EDA at doses of 30 and 45 mg/kg. Likewise, EDA significantly improved complex I and IV activity, apoptosis and necrosis, ROS, troponin I, and lactate levels following AlP-poisoning (*p* < 0.05). Also, the mean survival time was increased following EDA treatment, which can be attributed to the EDA’s protective effects against diverse underlying mechanisms of phosphine-induced cardiac toxicity. These findings suggest that EDA, by ameliorating heart function and modulating mitochondrial activity, might relieve AlP-induced cardiotoxicity. Nonetheless, additional investigations are required to examine any potential clinical advantages of EDA in this toxicity.

## 1 Introduction

The insecticide aluminum phosphide (AlP) is a solid fumigant known as a rice tablet in Iran. This pesticide is extensively utilized for agricultural product protection from insects and rodents during transportation and storage (Mehrpour et al., 2012). Despite the high toxicity of this pesticide for humans, it is still preferred for use by farmers because it is cost-beneficial, has high efficiency for insect control, and has a minimum residual on stored food (Anand et al., 2011; Aminjan et al., 2019). Despite all these advantages for managing insects and rodents, widespread evidence shows that AlP poisoning is potentially fatal for humans. It has been reported that more than 70% of poisoned patients die from AlP ingestion (Bogle et al., 2006; Anand et al., 2011). According to the Iranian Forensic Medicine Center reports, the number of deaths due to AlP poisoning has increased (Bagherian et al., 2021). Based on clinical trials, following ingestion, the AlP tablet’s contact with water or moisture, or hydrochloric acid in the stomach, leads to releasing an extremely toxic gas named phosphine. The exact mechanism of phosphine toxicity has not been known; nevertheless, based on some findings from animal studies, the intensive oxidative stress, disturbance of electron transport chain (ETC) in mitochondria, interference with several macromolecules, and apoptosis are plausible mechanisms in this toxicity (Mehrpour et al., 2012; Moghadamnia, 2012). Phosphine intoxication causes failure in multi organs. However, cardiovascular toxicity is the most important cause of death due to this poisoning. Electrocardiographic (ECG) abnormalities, histopathological changes, severe hypotension, left ventricle hypokinesia, and congestive heart failure occur because of phosphine intoxication (Bogle et al., 2006). So, to identify an effective treatment strategy, it is essential to focus on the cardiovascular consequences of phosphine toxicity (Karami-Mohajeri et al., 2013). Unfortunately, there is no specific antidote for managing the cardiac toxicity effects of AlP to date. The protective impacts of several agents such as cannabidiol (Hooshangi Shayesteh et al., 2022), acetyl-l-carnitine (Baghaei et al., 2016), vasopressin (Jafari et al., 2015), melatonin (Asghari et al., 2017), Mg nanoparticle (Baeeri et al., 2013), triiodothyronine (Abdolghaffari et al., 2015), iron sucrose (Solgi et al., 2015), levosimendan (Armandeh et al., 2021; Baeeri et al., 2022), taurine (Samadi et al., 2021), exenatide (Bameri et al., 2021), and minocycline (Haghi-Aminjan et al., 2018) on the cardiac toxicity of phosphine have been investigated. However, there is no entirely effective treatment for the management of AlP-cardiotoxicity.

Edaravone (EDA, Radicava) is a known potent free radical scavenger and strong antioxidant that has been clinically used to treat acute ischemic stroke and amyotrophic lateral sclerosis since 2001 and 2017, respectively (Kikuchi et al., 2013a; Cruz, 2018). Various studies have shown that edaravone has antioxidant effects because it can reduce hydroxyl radicals and hydroxyl radical-dependent lipid peroxidation (Watanabe et al., 1994; Yamamoto et al., 1997). Edaravone ameliorates vascular blood flow by increasing the expression of endothelial nitric oxide synthetase (eNOS) and reducing LDL oxidation (Yoshida et al., 2005). Recently, EDA can have protective effects against apoptosis and inflammation caused by Ca^+2^ overloads, ROS production, and iNOS expression in cardiovascular disease (Kikuchi et al., 2013b). EDA reduces cardiac biomarkers with potent antioxidant effects. It also has anti-apoptotic, anti-necrotic, and anti-inflammatory effects on cardiovascular diseases (Hassan et al., 2016). Also, EDA ameliorates the left ventricular ejection fraction (LVEF), reduces the serum concentration of creatine kinase-MB isoenzyme, and reduces the size of myocardial infarction (Tsujita et al., 2004; Tsujita et al., 2006; Nakamura et al., 2009). Furthermore, previous studies demonstrated the effectiveness of EDA treatment in several models of cardiotoxicity (Saibara et al., 2003; Xin et al., 2011; Emekli-Alturfan et al., 2015; Hassan et al., 2015; Koohsari et al., 2016; Basol et al., 2019).

The outcomes of investigations into the substantial protective effects on the cardiovascular system and the solid anti-apoptotic, antioxidant and antiarrhythmic effects of EDA bring about the idea that EDA could be a highly effective drug to improve phosphine-induced cardiotoxicity. Therefore, this research aimed to examine the potential protective effects of EDA on hemodynamic, electrocardiographic (ECG), echocardiographic, molecular, and biochemical properties in rats poisoned with aluminum phosphide at two specific time intervals.

## 2 Material and method

### 2.1 Chemicals

Edaravone was purchased from Sigma-Aldrich. The AlP tablets were supplied by Samiran Company (Iran). The Mitochondria isolation kit was obtained from BioChain Inc. (United States). The ApoFlowExVR fluorescein isothiocyanate (FITC) kit was purchased from Exbio (Vestec, Czech Republic). Kits for lactate and troponin I assay were purchased from ZellBio (Germany). The rest of the chemicals were supplied by Sigma-Aldrich (Germany).

### 2.2 Animals

Male Wistar rats weighing 200–250 g were utilized in the experiments. The animals were kept in standard polycarbonate cages in suitable conditions, including a 12-h light-dark cycle, a 20°C–25°C temperature, and a humidity of 50%. A normal diet and water were readily available to rats. All animal processes were done following the authorized, ethical protocols and guidelines for using animals of the Ethics Committee at TUMS (CO: IR.TUMS.MEDICINE.REC.1399.756).

### 2.3 Determination LD_50_ of AlP

In the previous studies, AlP LD_50_ was reported at 8.5–12.7 mg/kg. Usually, 100% mortality (LD_100_) occurs at doses above 14 mg/kg, and mortality is not observed in amounts less than 8 mg/kg. Therefore, to determine the precise LD_50_ of AlP for this study, doses of 8, 10, 12, and 14 mg/kg were examined in four groups (*n* = 4). For this purpose, the powdered AlP tablet was dissolved in almond oil and orally gavaged to the rats. After monitoring for 24 h, the mortality was recorded in each group. Finally, according to Karber’s method (Akhila et al., 2007), the LD_50_ was calculated as 11.5 mg/kg and was used for all experiments.

### 2.4 Study design

Initially, three EDA dosages of 20, 30, and 45 mg/kg were chosen based on the pilot trial results. In the first step, six groups, including twelve rats, were randomly defined to examine the ECG, hemodynamic, and echocardiographic parameters. The groups included: (Mehrpour et al., 2012): almond oil (control), (Anand et al., 2011), normal saline, (Aminjan et al., 2019), AlP (LD_50_), (Bogle et al., 2006), AlP+20 mg/kg of EDA, (Bagherian et al., 2021), AlP+30 mg/kg of EDA, and (Moghadamnia, 2012) AlP+45 mg/kg of EDA. Six rats in each group were utilized for ECG and hemodynamic parameter examination. Eventually, after 24 h, the six live rats in each group were utilized for echocardiographic examination. The solvents of EDA and AlP were normal saline and almond oil, respectively. The EDA was injected 1 h following the AlP poisoning intraperitoneally (IP). In the second step, for biochemical examinations, six groups similar to the aforesaid groups were followed for 12 h, and six other groups were observed for 24 h. Eventually, at 12 and 24 h after the treatment, six live rats were sacrificed in each group.

### 2.5 Assessment of hemodynamic and electrocardiogram parameters

To record the parameters, including PR interval, QRS complex duration, ST height, QTc, blood pressure (BP), and heart rate (HR), the PowerLab device (PowerLab 4/35 Data Acquisition Systems, AD Instruments, Australia) was utilized. At first, AlP was orally gavaged to all rats, excluding the control group. After 30 min, the rats, based on ketamine/xylazine (50/5 mg/kg), were anesthetized and connected to the PowerLab device utilizing the electrodes. EDA was injected 30 min after the connection, and the rats were monitored for 180 min. To maintain complete anesthesia, a maintenance dose of ketamine (25–30 mg/kg) was injected every 45 min. Also, the systolic blood pressure was measured utilizing a noninvasive BP tail cuff.

### 2.6 Echocardiography

After 24 h, the alive rats were anesthetized, using simultaneous ketamine 50 mg/kg and xylazine 5 mg/kg, and their chest hair was shaved. To examine the echocardiography parameters, including stroke volume (SV) and ejection fraction (EF), the M-mode short-axis echocardiography was accomplished by utilizing a cardiac ultrasound (GE Vivid seven Dimension Ultrasound, Horten, Norway). The cardiac output (CO) was calculated by multiplying the heart rate and stroke volume.

### 2.7 Heart tissue sampling

After following for 12 and 24 h, the rats were anesthetized with ketamine (50 mg/kg) and xylazine (5 mg/kg) and then sacrificed. The hearts of the rats were surgically isolated, and to wipe the blood, it was washed with cold phosphate buffer. Then, the heart tissues were frozen at −80°C for biochemical and mitochondrial assays.

### 2.8 Assessment of heart tissues protein concentration

The Bradford spectrophotometry protein assay assessed the heart tissue protein concentration. As the standard protein concentration, bovine serum albumin was utilized. The heart tissue samples’ absorbance values were determined at 595 nm using a spectrophotometer set setup (Bradford, 1976).

### 2.9 Measurement of mitochondrial complex I, II, IV activities

Investigation of complex I (NADH dehydrogenase) activity was performed based on the Sherwood and Hirst method (Sherwood and Hirst, 2006), which was formerly set up in our lab (Haghi-Aminjan et al., 2018). The results of NADH dehydrogenase activity were reported as nanomoles of NADH per minute per mg of protein. For determining the succinate dehydrogenase (Complex II) activity in heart tissue, we used the method reported by Tompkins et al. (2006). This procedure was formerly set up in our lab (Jafari et al., 2015). The results of succinate dehydrogenase activity were presented as nanomoles of 2-dichlorophenolindophenol (DCPIP) per min per mg of protein. Investigation of complex IV (cytochrome c oxidase) activity in heart tissue was conducted according to the Cooperstein and Lazarow method (Cooperstein and Lazarow, 1951), which was formerly set up in our lab (Baghaei et al., 2016). The cytochrome c oxidase activity results were presented as a K unit per minute per mg of protein.

### 2.10 Reactive oxygen species (ROS) measurement

The level of heart mitochondrial ROS was examined with a fluorescence spectrophotometer utilizing 2′,7′-dichlorofluorescein diacetate (DCF-DA), which cellular peroxides changed into highly fluorescent DCF. At 37°C and in a dark environment, the supernatants were incubated with 5 µM DCF-DA for 30 min. A fluorometer was then used to record fluorescence at 525 nm of emission and 488 nm of excitation. As a result, the ROS level in each group was assessed using a standard curve and expressed as Unit/mg of tissue protein (Moeini-Nodeh et al., 2017).

### 2.11 Investigation of apoptosis and necrosis applying flow cytometry

Initially, according to the method of Schlüter, the cardiac cells were separated from fresh cardiac tissue (Schlüter and Schreiber, 2005). Then cardiomyocytes were stained according to a FITC-annexin V/PI kit protocol. Finally, to analyze the percentages of apoptosis and necrosis cells, the specimens were injected into the Mindray flow cytometer (Mindray, Shenzhen, China). Utilizing the Apogee histogram software, the data were investigated (Rahimifard et al., 2020).

### 2.12 Cardiac tissue injury markers investigation

The lactate level of heart tissue was measured on a homogenous tissue according to the lactate assay kit protocol (ZellBio GmbH, Germany). The troponin I (cTnI) level was assessed on homogenated heart tissue according to the kit protocol of ZellBio GmbH, Germany. To check the absorbance of specimens, a microplate reader (Synergy, BioTek Instruments Inc., Germany) was utilized.

### 2.13 Assessment of survival time

A survival time assessment was conducted utilizing four groups, each including six rats as follows: (Mehrpour et al., 2012): received AlP (LD_50_); (Anand et al., 2011); AlP+20 mg/kg of EDA; (Aminjan et al., 2019); AlP+30 mg/kg of EDA; (Bogle et al., 2006); AlP+45 mg/kg of EDA. Each group’s number of dead rats was recorded after a 48-h follow-up.

### 2.14 Statistical analysis

The Kaplan-Meier method with log-rank (Mantel-Cox) was used as a survival time assessment method. A two-way ANOVA test was utilized to analyze the flow cytometry and ECG results. The one-way ANOVA test was conducted for data analysis of the other results. The Tukey test was used for the post hoc test, and *p* < 0.05 was regarded as statistically significant. The results are presented as mean ± standard deviation (SD).

## 3 Results

There were no significant differences between the almond oil and normal saline groups. Therefore, the data of the normal saline group are not shown.

### 3.1 Heart rate, blood pressure, and electrocardiographic parameters

The hemodynamic and electrocardiographic abnormalities, including hypotension, decreased heart rate, PR interval prolongation, widened QRS duration, QTc interval prolongation, and elevated ST height, following AlP administration were observed. The PR interval at 60–90, 120–150, and 150–180 min were significantly reduced with EDA treatment at 30 and 45 mg/kg dosages compared to the AIP group (*p* < 0.05). The QRS duration widening appeared 60–90 min after AlP treatment. EDA treatment in all three groups (AlP + EDA 20, AlP + EDA 30, and AlP + EDA 45) significantly improved the QRS duration at 120–150 and 150–180 min (*p* < 0.05). Further, QTc interval prolongation caused by AlP was significantly (*p* < 0.05) improved by 30 and 45 mg/kg of EDA treatment from 60 min onwards. A significant increase in ST-segment was observed in the AlP group compared to the control group. The EDA treatment (30 and 45 mg/kg) significantly reduced the ST segment in comparison to the Alp group after 60 min (*p* < 0.05) (Table 1). Also, compared to the control group, the HR and BP significantly decreased from 30 to 60 min onwards in phosphine-poisoned rats (*p* < 0.05). The progressive deterioration in BP due to AlP was improved with all doses from 90 to 120 min onwards (*p* < 0.05) (Table 2). Also, the EDA treatment significantly enhanced the HR reduction at 60–120 min at a 45 mg/kg dose, 120–150 min with a dose of 30 and 45 mg/kg, and 90–120 min onwards with all doses (Table 3).

**TABLE 1:** Electrocardiogram parameters variations in diverse groups.

Variable	Group	Time (min)					
		0–30	30–60	60–90	90–120	120–150	150–180
PR Interval (ms)	Control	43.7 ± 2.39	43.5 ± 2.77	44.3 ± 2.93	43.3 ± 2.13	43.7 ± 2.25	44.5 ± 2.33
	AlP (LD_50_)	45.2 ± 2.31	47.8 ± 1.88	50.7 ± 1.40^a^	57.4 ± 4.87^a^	63.0 ± 2.28^a^	62.5 ± 2.27^a^
	AlP + EDA 20	44.8 ± 2.16	46.5 ± 2.84	49.5 ± 1.97^a^	55.5 ± 1.76^a^	58.8 ± 2.56^a^	56.8 ± 2.39^a,b^
	AlP + EDA 30	44.7 ± 1.87	46.2 ± 1.89	47.1 ± 1.97 ^b^	50.4 ± 1.58^a^	52.4 ± 1.33^a,b^	52.9 ± 1.34^a,b^
	AlP + EDA 45	44.6 ± 1.96	45.3 ± 1.87	46.3 ± 1.82 ^b^	49.4 ± 1.33^a^	49.7 ± 1.32^a,b^	51.8 ± 1.79^a,b^
QRS duration (ms)	Control	15.5 ± 1.50	16.1 ± 2.18	14.9 ± 1.77	16.3 ± 1.63	15.6 ± 1.94	16.4 ± 0.49
	AlP (LD_50_)	16.2 ± 0.98	19.4 ± 1.37	20.9 ± 0.92^a^	20.9 ± 1.61^a^	25.4 ± 1.00^a^	28.1 ± 0.82^a^
	AlP + EDA 20	16.3 ± 1.13	19.2 ± 0.91	20.6 ± 1.42^a^	22.0 ± 1.53^a^	21.2 ± 1.34^a,b^	25.7 ± 1.53^a^
	AlP + EDA 30	16.9 ± 0.87	18.2 ± 0.84	18.5 ± 1.98	20.3 ± 1.19^a^	20.2 ± 1.22^a,b^	20.4 ± 1.08^a,b^
	AlP + EDA 45	16.0 ± 0.61	17.2 ± 0.85	18.6 ± 1.32^a^	19.2 ± 0.67^a^	19.3 ± 1.57^a,b^	20.2 ± 1.43^a,b^
QTc (ms)	Control	111.3 ± 8.90	124.0 ± 14.3	125.3 ± 6.90	130.6 ± 8.72	121.4 ± 12.9	117.6 ± 9.29
	AlP (LD_50_)	128.3 ± 6.20^a^	146.0 ± 14.9	172.7 ± 14.8^a^	172.3 ± 15.7^a^	203.3 ± 13.6^a^	226.1 ± 5.27^a^
	AlP + EDA 20	121.2 ± 10.0	131.0 ± 7.60	141.5 ± 9.44 ^b^	151.4 ± 13.8	164.5 ± 15.2^a,b^	184.8 ± 21.5^a,b^
	AlP + EDA 30	120.8 ± 12.8	128.0 ± 12.3	130.3 ± 12.5 ^b^	140.7 ± 14.7 ^b^	145.0 ± 16.8 ^b^	172.0 ± 6.90^a,b^
	AlP + EDA 45	121.3 ± 7.54	124.5 ± 5.92	131.3 ± 6.46 ^b^	143.4 ± 17.3	136.3 ± 8.22 ^b^	133.3 ± 13.4 ^b^
ST Height (µv)	Control	42.16 ± 3.15	47.56 ± 3.59	44.97 ± 8.61	45.18 ± 4.37	39.26 ± 6.04	45.44 ± 7.12
	AlP (LD_50_)	57.56 ± 8.14^a^	64.51 ± 5.83^a^	142.6 ± 8.72^a^	148.5 ± 18.2^a^	169.5 ± 20.9^a^	167.4 ± 15.4^a^
	AlP + EDA 20	47.48 ± 4.16	61.01 ± 4.96^a^	130.0 ± 10.1^a^	142.8 ± 5.99^a^	144.8 ± 6.67^a^	159.8 ± 8.17^a^
	AlP + EDA 30	42.51 ± 4.89^a^	68.90 ± 6.53^a^	114.1 ± 11.7^a,b^	128.7 ± 7.64^a^	119.0 ± 8.64^a,b^	136.6 ± 16.1^a^
	AlP + EDA 45	44.45 ± 9.12	72.12 ± 8.20^a^	88.79 ± 9.10^a,b^	93.85 ± 10.1^a,b^	95.07 ± 4.21^a,b^	100.2 ± 13.2^a,b^

The data is demonstrated as the mean ± standard deviation (*n* = 6). The almond oil was gavaged to the control group; the AlP (LD_50_) group was gavaged with 11.5 mg/kg of aluminum phosphide; the AlP + EDA, 20, AlP + EDA, 30, and AlP + EDA, 45 groups were given AlP (11.5 mg/kg) and 20, 30, and 45 mg/kg of EDA (IP), respectively. EDA: edaravone; AlP: Aluminum phosphide. ^a^ *p* < 0.05 *versus* the control group. ^b^ *p* < 0.05 *versus* the AlP (LD_50_) group.

**TABLE 2:** Blood pressure variations in diverse groups.

Group	Time (min)					
	0–30	30–60	60–90	90–120	120–150	150–180
Control	95.7 ± 3.9	98.5 ± 4.5	94.3 ± 5.05	97.7 ± 3.9	96.8 ± 2.6	97.7 ± 2.9
AlP (LD_50_)	92 ± 3.6	84.7 ± 5.5^a^	64.5 ± 6.41^a^	52.7 ± 4.8^a^	50.2 ± 4.2^a^	49.3 ± 4.3^a^
AlP + EDA 20	93.5 ± 5.2	86.8 ± 3.8^a^	70.2 ± 3.87^a^	67.3 ± 4.5^a,b^	69.7 ± 5.5^a,b^	64.2 ± 4.4^a,b^
AlP + EDA 30	91 ± 7	83.8 ± 6.7^a^	68.7 ± 3.98^a^	75.2 ± 11^a,b^	73 ± 7^a,b^	69 ± 5.2^a,b^
AlP + EDA 45	91.2 ± 4.4	86.8 ± 8.2	71.7 ± 3.2^a^	77 ± 3.2^a,b^	83.5 ± 3.6^a,b^	83.7 ± 5.2^a,b^

The data is demonstrated as the mean ± standard deviation (*n* = 6). The almond oil was gavaged to the control group; the AlP (LD_50_) group was gavaged with 11.5 mg/kg of aluminum phosphide; the AlP + EDA, 20, AlP + EDA, 30, and AlP + EDA, 45 groups were given AlP (11.5 mg/kg) and 20, 30, and 45 mg/kg of EDA (IP), respectively. EDA: edaravone; AlP: Aluminum phosphide. ^a^ *p* < 0.05 *versus* the control group. ^b^ *p* < 0.05 *versus* the AlP (LD_50_) group.

**TABLE 3:** Heart rate variations in diverse groups.

Group	Time (min)					
	0–30	30–60	60–90	90–120	120–150	150–180
Control	324.5 ± 8.3	324 ± 11	316 ± 9.2	324.3 ± 9.3	322 ± 9.3	323.7 ± 8.7
AlP (LD_50_)	308.3 ± 9.5	262.7 ± 12^a^	236.8 ± 8.5^a^	229 ± 14^a^	197.7 ± 19^a^	169.3 ± 9.7^a^
AlP + EDA 20	308.2 ± 8.3	266 ± 14^a^	239.8 ± 11^a^	241.3 ± 16^a^	232.2 ± 10^a^	214 ± 6.1^a,b^
AlP + EDA 30	307 ± 12	272.5 ± 12^a^	252 ± 14^a^	238.8 ± 13^a^	241.8 ± 9.3^a,b^	73.3 ± 9.8^a,b^
AlP + EDA 45	307.3 ± 13	274.5 ± 6.2^a^	260 ± 13^a,b^	263.3 ± 11^a,b^	252.5 ± 8.6^a,b^	257.5 ± 7.2^a,b^

The data is demonstrated as the mean ± standard deviation (*n* = 6). The almond oil was gavaged to the control group; the AlP (LD_50_) group was gavaged with 11.5 mg/kg of aluminum phosphide; the AlP + EDA, 20, AlP + EDA, 30, and AlP + EDA, 45 groups were given AlP (11.5 mg/kg) and 20, 30, and 45 mg/kg of EDA (IP), respectively. EDA: edaravone; AlP: Aluminum phosphide. ^a^ *p* < 0.05 *versus* the control group. ^b^ *p* < 0.05 *versus* the AlP (LD_50_) group.

### 3.2 Ejection fraction, stroke volume, and cardiac output

**FIGURE 1:**
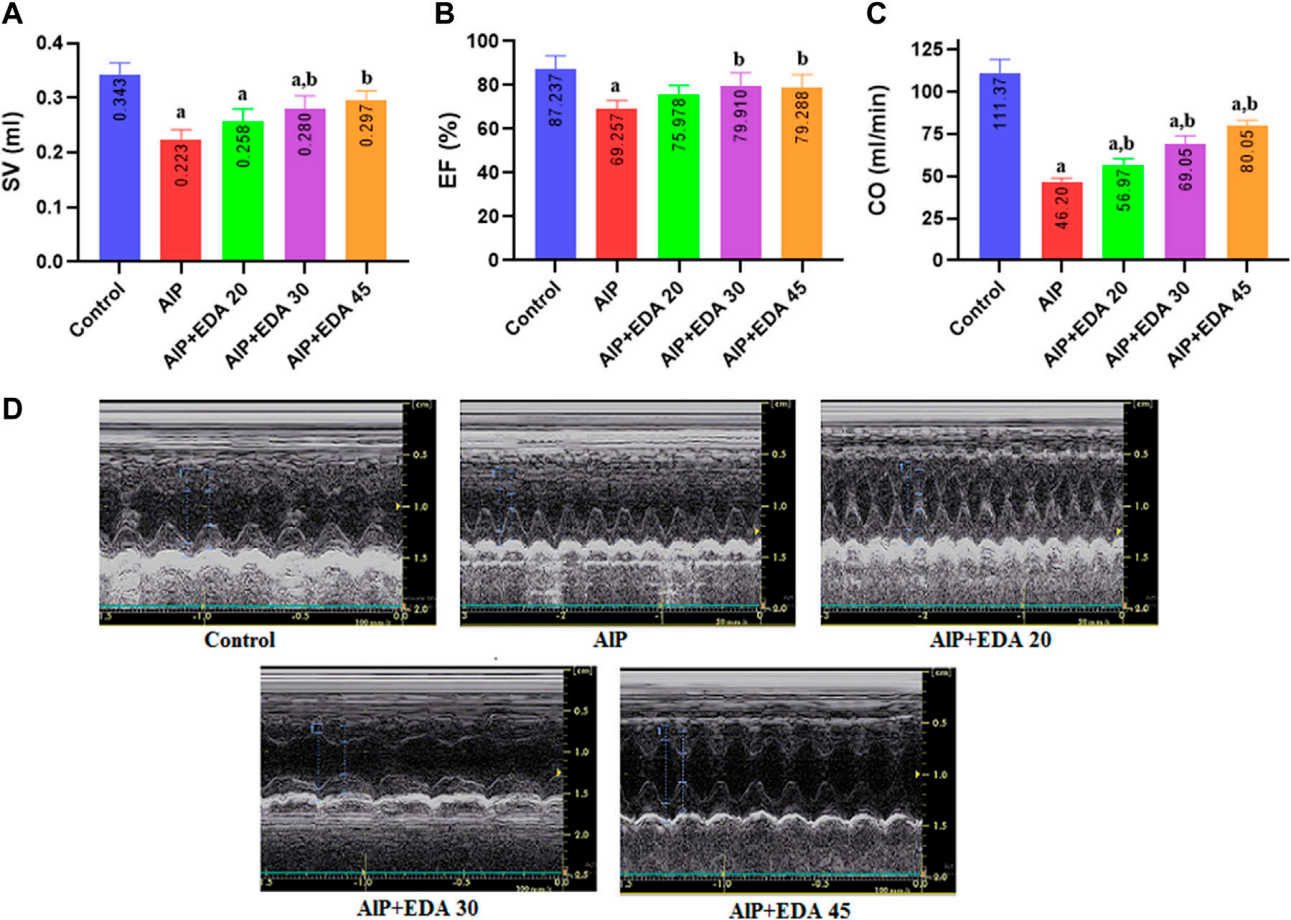
The impact of various treatments on LVEF%, SV, and CO. **(A)** Left ventricular Stroke volume; **(B)** Left ventricular ejection fraction (EF%); **(C)** Cardiac output (CO); **(D)** Illustrations of echocardiography from the cardiac function of each group (recorded for 3 min). The data is demonstrated as the mean ± standard deviation (*n* = 6). The almond oil was gavaged to the control group; the AlP (LD_50_) group was gavaged with 11.5 mg/kg of aluminum phosphide; the AlP + EDA 20, AlP + EDA 30, and AlP + EDA 45 groups were given AlP (11.5 mg/kg) and 20, 30, and 45 mg/kg of EDA (IP), respectively. EDA: Edaravone; AlP: Aluminum phosphide ^a^ *p* < 0.05 *versus* the control group. ^b^ *p* < 0.05 *versus* the AlP (LD_50_) group.

The findings showed that AlP poisoning significantly impaired the systolic function of the heart by reducing the SV, LVEF, and CO (*p* < 0.05). The SV and LVEF significantly improved with EDA treatment at 30 and 45 mg/kg dosages (*p* < 0.05). Also, the cardiac output was significantly improved compared to the AlP group in all three EDA doses (20, 30, and 45 mg/kg). In the evaluation of EF, the difference between the control group and EDA treatment at 30 and 45 mg/kg dosages was not significant (*p* < 0.05). In addition, in the SV evaluation, the difference between the control group and the AlP + EDA 45 mg/kg group was not significant (*p* < 0.05) (Figure 1).

### 3.3 Mitochondrial respiratory complex activity

#### 3.3.1 Activity of complex I

The mitochondrial complex I activity was significantly decreased in the AlP poisoning groups at 12 and 24 h (*p* < 0.05). As shown in Table 4, treatment with 30 and 45 mg/kg doses of EDA in the 24 h period and 45 mg/kg in the 12 h period significantly improved the complex I activity compared to the relevant AIP group (*p* < 0.05).

**TABLE 4:** Effect of treatments on mitochondrial complexes activity.

Groups	Time(hour)	Pharameters		
		Complex I activity (nmol/min/mg protein)	Complex II activity (nmol/min/mg protein)	Complex IV activity (K/min/mg protein)
Control	12 h	314.33 ± 8.177	85.56 ± 9.87	464.47 ± 5.74
AlP (LD_50_)		211.91 ± 14.49^a^	79.26 ± 13.13	318.89 ± 14.35^a^
AlP + EDA 20		222.07 ± 7.866^a^	81.19 ± 11.47	335.01 ± 9.99^a^
AlP + EDA 30		225.39 ± 10.24^a^	82.73 ± 13.78	363.10 ± 13.13^a,b^
AlP + EDA 45		244.44 ± 7.217^a,b^	81.16 ± 9.59	404.09 ± 6.35^a,b^
Control	24 h	316.99 ± 9.705	86.38 ± 7.21	465.35 ± 8.69
AlP (LD_50_)		192.69 ± 12.26^a^	82.33 ± 12.38	296.6 ± 11.54^a^
AlP + EDA 20		208.10 ± 9.286^a^	80.23 ± 11.65	312.39 ± 9.32^a^
AlP + EDA 30		219.26 ± 5.658^a,b^	82.89 ± 6.60	396.54 ± 8.10^a,b^
AlP + EDA 45		263.25 ± 9.624^a,b^	83.83 ± 11.40	440.79 ± 8.89^a,b^

The data is demonstrated as the mean ± standard deviation (*n* = 6). The almond oil was gavaged to the control group; the AlP (LD_50_) group was gavaged with 11.5 mg/kg of aluminum phosphide; the AlP + EDA, 20, AlP + EDA, 30, and AlP + EDA, 45 groups were given AlP (11.5 mg/kg) and 20, 30, and 45 mg/kg of EDA (IP), respectively. EDA: edaravone; AlP: Aluminum phosphide. ^a^ *p* < 0.05 *versus* the control group.^b^ *p* < 0.05 *versus* the AlP (LD_50_) group.

#### 3.3.2 Complex II activity

There were no significant differences in complex II activity between the treated groups and the control group (*p* < 0.05) (Table 4).

#### 3.3.3 Complex IV activity

AlP poisoning significantly repressed the activity of complex IV at 12 and 24 h duration compared to the control groups (*p* < 0.05). Treatment with 30 and 45 mg/kg doses of EDA significantly improved the complex IV activity compared to the relevant AIP group in 24 and 12 h times. (*p* < 0.05) (Table 4).

### 3.6 Reactive oxygen species in heart tissue

**FIGURE 2:**
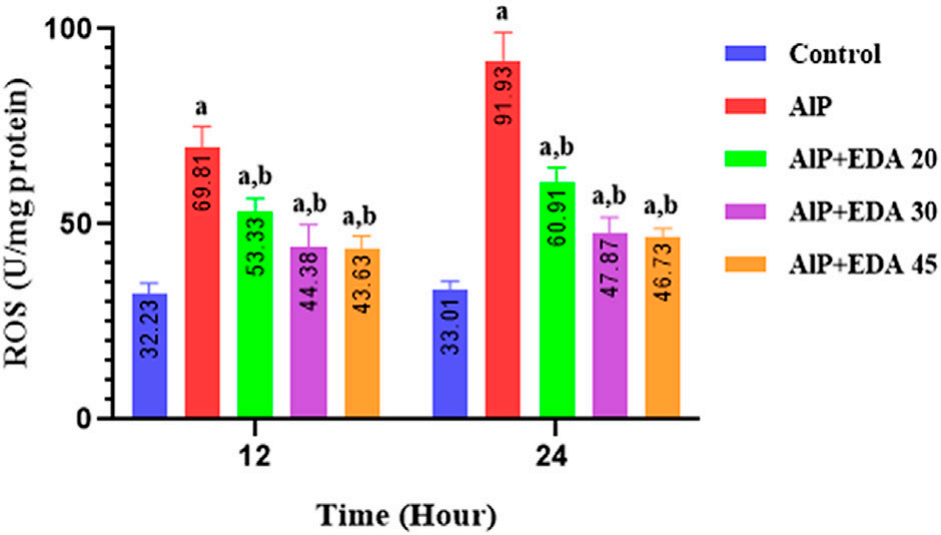
Effect of treatments on reactive oxygen species (ROS) in the cardiac tissue of rats. The data is demonstrated as the mean ± standard deviation (*n* = 6). The almond oil was gavaged to the control group; the AlP (LD_50_) group was gavaged with 11.5 mg/kg of aluminum phosphide; the AlP + EDA 20, AlP + EDA 30, and AlP + EDA 45 groups were given AlP (11.5 mg/kg) and 20, 30, and 45 mg/kg of EDA (IP), respectively. EDA: Edaravone; AlP: Aluminum phosphide ^a^ *p* < 0.05 *versus* the control group. ^b^ *p* < 0.05 *versus* the AlP (LD_50_) group.

The heart mitochondrial ROS level was significantly enhanced by AlP poisoning (*p* < 0.05). As shown in Figure 2, administration of EDA in all doses (20, 30, and 45 mg/kg) significantly reduced the ROS level in the heart tissue compared to the relevant AIP group at both 24 and 12 h times (*p* < 0.05).

### 3.7 Apoptosis and necrosis

**FIGURE 3:**
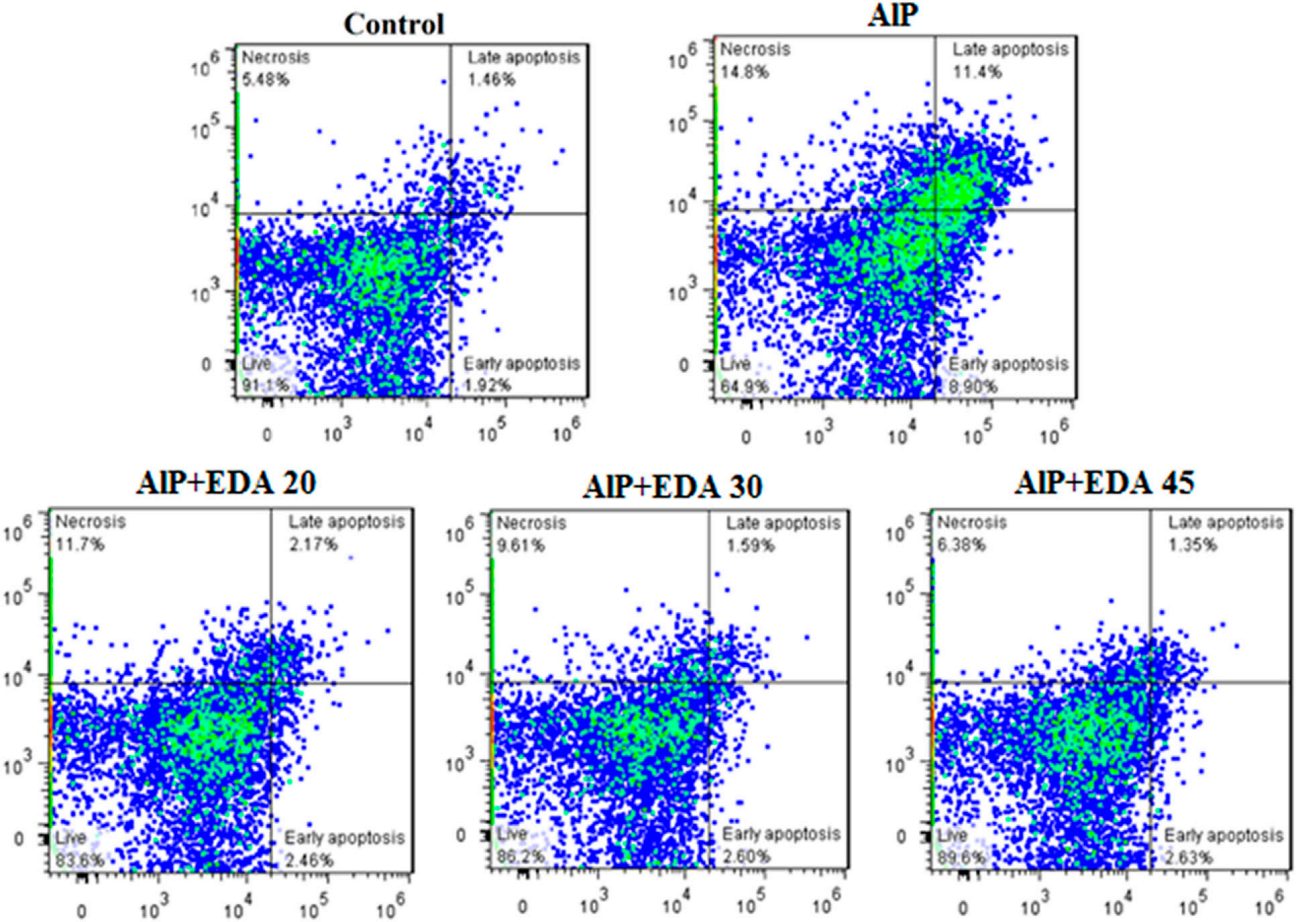
Images of flow cytometry of the cardiomyocytes in different groups. The utilization of annexin-V/PI staining for the examination of the cell death mode. The almond oil was gavaged to the control group; the AlP (LD_50_) group was gavaged with 11.5 mg/kg of aluminum phosphide; the AlP + EDA 20, AlP + EDA 30, and AlP + EDA 45 groups were given AlP (11.5 mg/kg) and 20, 30, and 45 mg/kg of EDA (IP), respectively. EDA: Edaravone; AlP: Aluminum phosphide.

**FIGURE 4:**
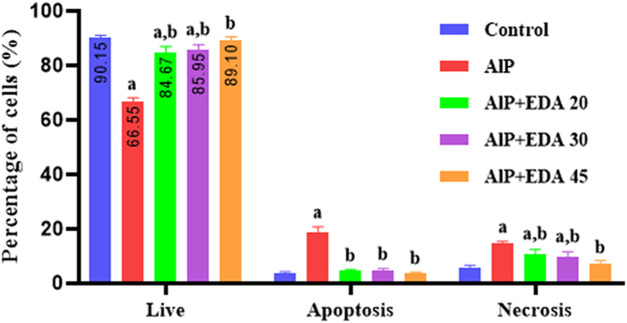
Analysis of flow cytometry of the live/apoptotic/necrotic cells. The data is demonstrated as the mean ± standard deviation (*n* = 6). The almond oil was gavaged to the control group; the AlP (LD_50_) group was gavaged with 11.5 mg/kg of aluminum phosphide; the AlP + EDA 20, AlP + EDA 30, and AlP + EDA 45 groups were given AlP (11.5 mg/kg) and 20, 30, and 45 mg/kg of EDA (IP), respectively. EDA: Edaravone; AlP: Aluminum phosphide ^a^ *p* < 0.05 *versus* the control group. ^b^ *p* < 0.05 *versus* the AlP (LD_50_) group.

The flow cytometry analysis shows the percentage of live cells, late apoptotic cells, early apoptotic cells, and necrotic cells (Figure 3). The live cells percentage in the control group was 91.1%. AlP poisoning significantly decreased the viability of heart cells to 64.9%, increasing the percentage of apoptosis and necrosis cells compared to the control group (*p* < 0.05). The EDA treatment in a dose-dependent procedure significantly enhanced live cells from 64.9% up to 83.6%, 86.2%, and 89.6%, utilizing 20, 30, and 45 mg/kg of EDA, respectively (*p* < 0.05). In the same way, the early apoptosis, late apoptosis, and necrosis cells significantly decreased in a dose-dependent manner utilizing 20, 30, and 45 mg/kg of EDA. It should be noted that the difference between the percentage of live cells in the control group and the AlP + EDA 45 mg/kg group was not significant (Figure 4).

### 3.8 Lactate level

**FIGURE 5:**
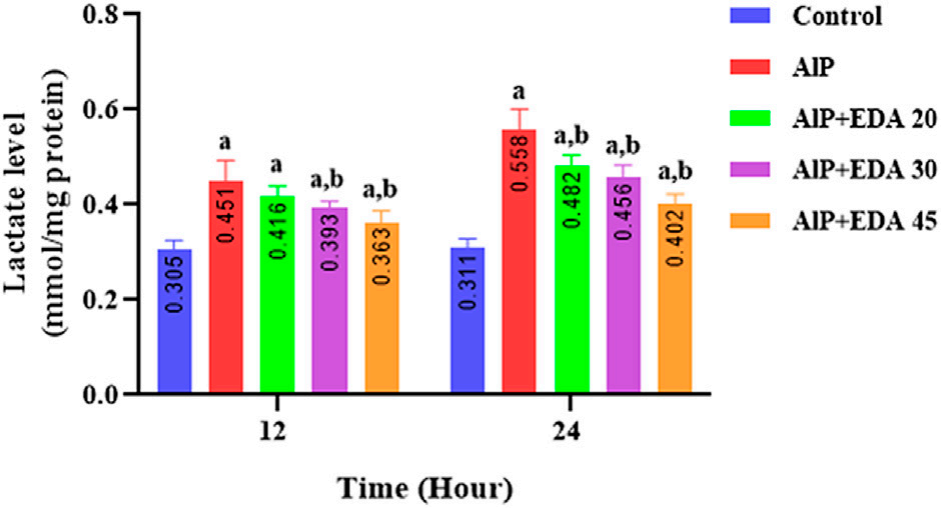
Effect of treatments on rat cardiac tissue lactate level. The data is demonstrated as the mean ± standard deviation (*n* = 6). The almond oil was gavaged to the control group; the AlP (LD_50_) group was gavaged with 11.5 mg/kg of aluminum phosphide; the AlP + EDA 20, AlP + EDA 30, and AlP + EDA 45 groups were given AlP (11.5 mg/kg) and 20, 30, and 45 mg/kg of EDA (IP), respectively. EDA: Edaravone; AlP: Aluminum phosphide ^a^ *p* < 0.05 *versus* the control group. ^b^ *p* < 0.05 *versus* the AlP (LD_50_) group.

As shown in Figure 5, in the AlP groups, the cardiac tissue lactate levels were significantly increased compared to the control groups at 12 and 24 h (*p* < 0.05). Treatment with 20, 30, and 45 mg/kg doses of EDA in the 24 h duration, as well as 30 and 45 mg/kg in the 12 h duration, significantly decreased the heart tissue lactate level in comparison to the relevant AIP group (*p* < 0.05) (Figure 5).

### 3.11 Troponin-I level

**FIGURE 6:**
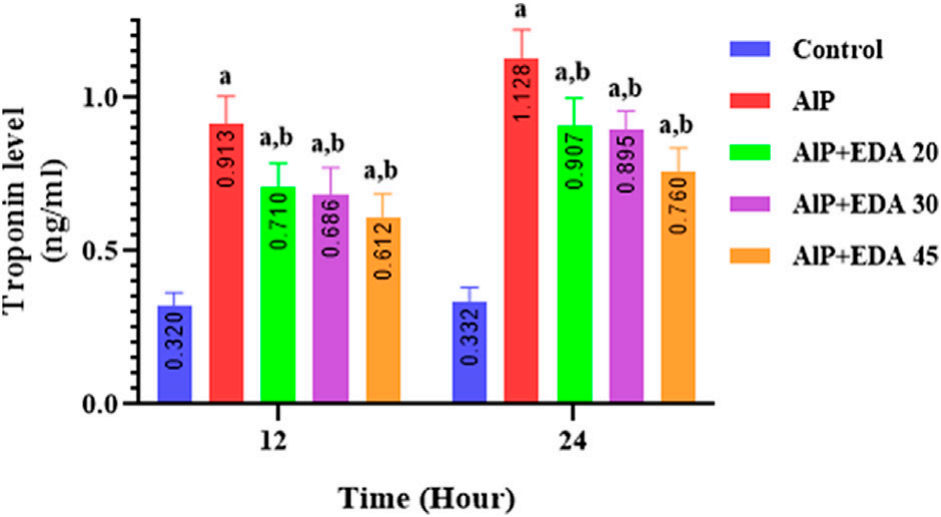
Effect of treatments on rat cardiac tissue cTnI level. The data is demonstrated as the mean ± standard deviation (*n* = 6). The almond oil was gavaged to the control group; the AlP (LD_50_) group was gavaged with 11.5 mg/kg of aluminum phosphide; the AlP + EDA 20, AlP + EDA 30, and AlP + EDA 45 groups were given AlP (11.5 mg/kg) and 20, 30, and 45 mg/kg of EDA (IP), respectively. EDA: Edaravone; AlP: Aluminum phosphide ^a^ *p* < 0.05 *versus* the control group. ^b^ *p* < 0.05 *versus* the AlP (LD_50_) group.

The heart tissue cTnI level was significantly enhanced by AlP poisoning at both times (12 and 24 h) in comparison to the control group (*p* < 0.05). As shown in Figure 6, administration of EDA in all doses significantly reduced the heart tissue cTnI level at 12 and 24 h times compared to the relevant AIP group (*p* < 0.05).

### 3.12 Survival time

**FIGURE 7:**
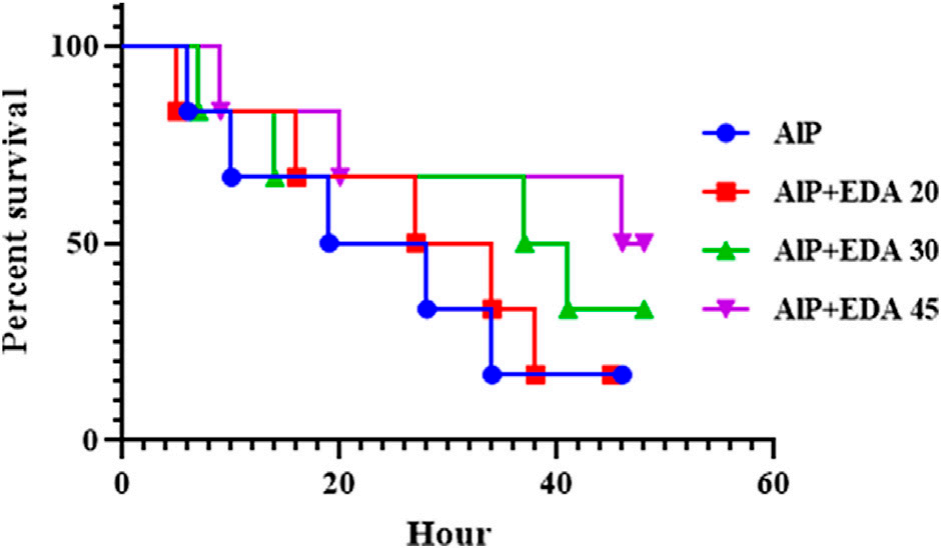
Kaplan-Meier survival curve analysis (*n* = 6). The almond oil was gavaged to the control group; the AlP (LD_50_) group was gavaged with 11.5 mg/kg of aluminum phosphide; the AlP + EDA 20, AlP + EDA 30, and AlP + EDA 45 groups were given AlP (11.5 mg/kg) and 20, 30, and 45 mg/kg of EDA (IP), respectively. EDA: Edaravone; AlP: Aluminum phosphide.

After 48 h of monitoring of groups, the survival time for each group was determined. The median survival time for the rats poisoned with AlP (LD50) was 23.5 h. The median survival times for the AlP + EDA 20 mg/kg, AlP + EDA 30 mg/kg, and AlP + EDA 45 mg/kg groups were 30.5, 39, and 47 h, respectively. Also, the survival percentage for the AlP (LD_50_), AlP + EDA 20 mg/kg, AlP + EDA 30 mg/kg, and AlP + EDA 45 mg/kg groups were 16.66%, 16.66%, 33.33%, and 50%, respectively. This indicates that the chance of survival increases with an increasing dose of EDA (Figure 7).

## 4 Discussion

The current *in-vivo* study’s goal was to assess the effects of edaravone medication on phosphine-induced acute cardiac toxicity. As a potent free radical scavenger, previous studies have shown that EDA has powerful antioxidant effects that reduce oxidative stress, inflammation, apoptosis, necrosis, swelling, and lipid peroxidation. Also, it can improve heart function and increase mitochondrial activity. In addition, due to its low molecular weight and lipophilicity, EDA is easily absorbed by the tissue (Watanabe et al., 1994; Crompton, 1999). It is so effective that it can inhibit cerebral edema with a single intravenous injection in animal models (Folland et al., 1979; Abe et al., 1988; Nishi et al., 1989). Accordingly, EDA can be highly effective in improving AlP-induced cardiotoxicity (Tsujita et al., 2004; Kikuchi et al., 2013b; Hassan et al., 2016). This is the first study that used EDA’s protective effects on AlP-induced cardiotoxicity at two specific time intervals.

**FIGURE 8:**
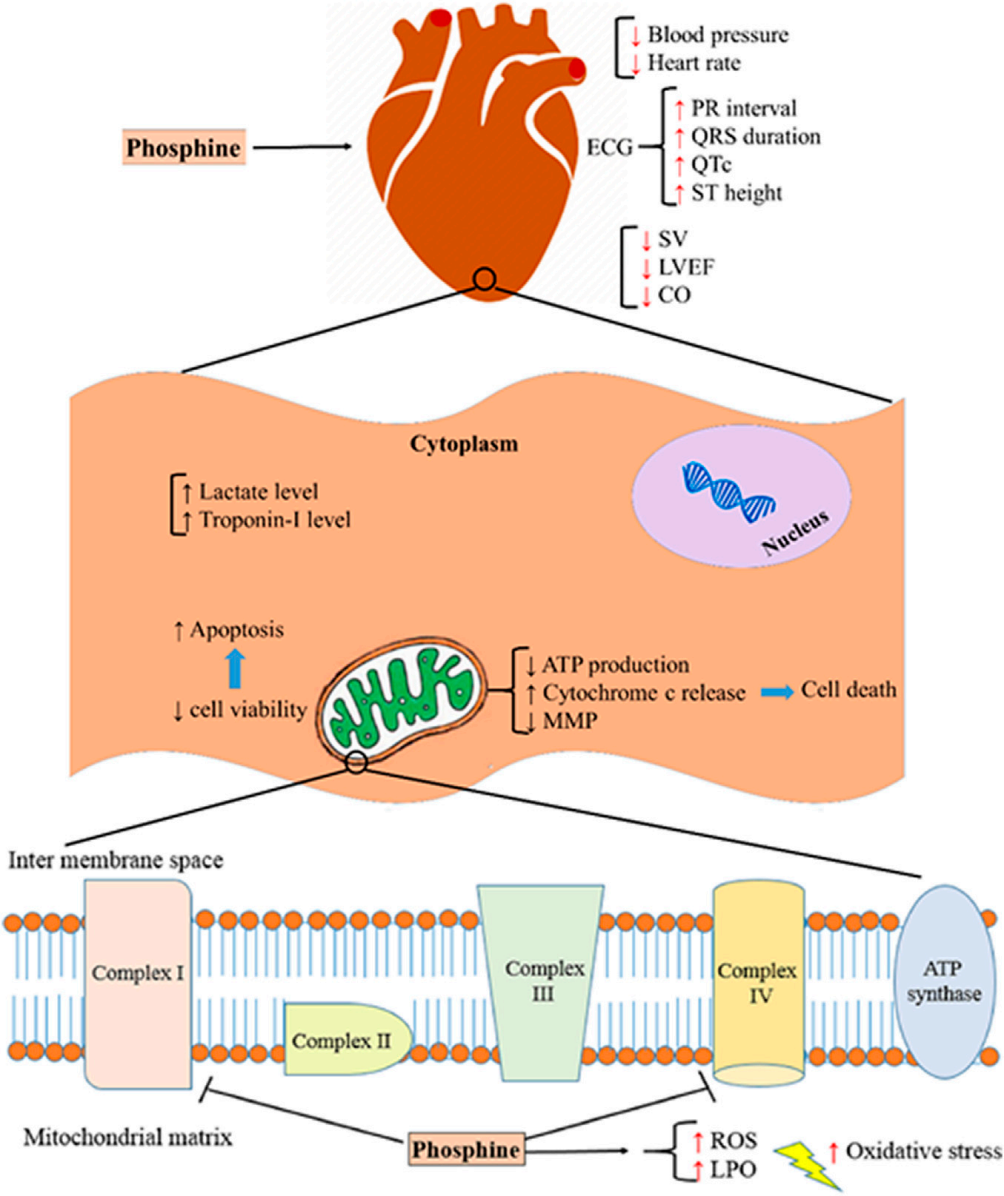
Aluminum phosphide cardiotoxicity mechanisms. SV; stroke volume, LVEF; left ventricular ejection fraction, CO; cardiac output, ATP; adenosine triphosphate, MMP; mitochondrial membrane potential, ROS; reactive oxygen species, LPO; lipid peroxidation.

Several studies have shown that cardiovascular disorders are the leading cause of death in the first 12–24 h after phosphine poisoning (Jafari et al., 2015; Asghari et al., 2017; Bameri et al., 2021). Based on some findings from previous studies, intensive oxidative stress, disturbance of electron transport chain (ETC) in mitochondria, interfering with several macromolecules, and apoptosis are the main mechanisms of AlP poisoning (Armandeh et al., 2021; Hooshangi Shayesteh et al., 2022). Figure 8 summarizes the mechanisms associated with phosphine cardiotoxicity. This study showed that AlP poisoning led to hemodynamic and electrocardiographic abnormalities, including hypotension, decreased heart rate, PR interval prolongation, widened QRS duration, QTc interval prolongation, and elevated ST height. Increased mortality rates in poisoned patients can be due to severe hypotension and decreased heart rate (Moghadamnia, 2012).

One of the study’s key findings was that the gradual deterioration in BP and bradycardia due to AlP was partially improved with the dose-dependent manner of EDA treatment. However, EDA-treated rats’ heart rate and blood pressure were not similar to non-poisoned rats. It seems this effect It seems this effect on the blood pressure and heart rate induced by EDA may be owing to its membrane-stabilizing property, maintenance of membrane integrity (Kikuchi et al., 2013b) and also retaining sufficient energy levels in cardiomyocytes. Edaravone does this with its powerful effect in inhibiting free radicals and lipid peroxidation and preventing the opening of mitochondrial permeability transition pores and the release of cytochrome C (Watanabe et al., 1994; Crompton, 1999). The PR interval, which indicates the beginning of atrial depolarization to the onset of ventricular depolarization, was prolonged due to AlP poisoning. EDA treatment reduced the PR interval after 1 h and improved atrioventricular conduction. The ST segment demonstrated that the interval between the end of ventricular depolarization and the outset of repolarization (Shah et al., 2009). Acute ST-elevation myocardial infarction is caused by transmural myocardial ischemia, which leads to myocardial damage or necrosis (Antman et al., 2000). The ST segment was elevated due to the AlP poisoning. Our findings revealed that this disorder, which indicates pericardial and myocardial damage, can be partially improved by EDA treatment. The QRS complex corresponds to the depolarization of the heart’s ventricles (Risk et al., 2005). The QT interval corresponds to the action potential duration and includes the duration of the ventricular depolarization and repolarization (Candil and Luengo, 2008). The QT interval is altered by acute myocardial ischemia, which also prolongs the maximal electrocardiographic QT interval and increases repolarization heterogeneity (represented as an increase in QT dispersion) (Bijl and Verheugt, 1992). Moreover, ventricular arrhythmias positively associated with the prolonged QTc interval (Wattoo et al., 2020). The duration of the QRS complex and QTc interval were increased by AlP poisoning, and treatment with EDA reversed these disorders significantly in poisoned rats. These effects on the hemodynamic and electrocardiogram induced by EDA may be owing to its membrane-stabilizing property and Ca^+2^ channel blockage effect (Kikuchi et al., 2013b). Xin et al. (2011) showed that the elevation of ST-segment and duration of QT interval due to doxorubicin was decreased by EDA, which can be due to the suppression of ROS and lipid peroxidation or the elimination of free radicals indirectly by activating antioxidant enzyme systems in tissues. One study reported that EDA post-treatment significantly reduced the conduction disturbances (increased QRS complex, increased QT interval, and increased ST-segment amplitude) due to amitriptyline-induced cardiotoxicity (Basol et al., 2019).

Studies have shown that AlP intoxication causes severe heart dysfunction, such as left ventricular hypokinesia or reduced ejection fraction (Gupta et al., 1995; Akkaoui et al., 2007; Mohan et al., 2016). Decreased EF owing to AlP poisoning causes heart shock, which can significantly cause severe hypotension due to loss of vascular integrity (Mehrpour et al., 2012). The current study’s findings showed that AlP poisoning significantly impaired the systolic function of the heart by reducing the SV, LVEF, and CO. This study showed that treatment with EDA significantly improved SV, LVEF, and CO. It should be noted that EDA treatment at 30 and 45 mg/kg dosages increased the LVEF near to that of the control group. In this regard, previous studies have indicated that EDA improves vascular blood flow by increasing the expression of endothelial nitric oxide synthetase (eNOS), improves the left ventricular ejection fraction, and reduces myocardial infarction size (Tsujita et al., 2004; Yoshida et al., 2005; Tsujita et al., 2006; Nakamura et al., 2009).

Previous studies have shown that cytochrome c oxidase (complex IV) is the leading site of the electron transport chain, which is disrupted by phosphine and reduces the level of ATP and energy required by the cell. Phosphine appears to be a nonspecific inhibitor of cytochrome and interacts with any macromolecule and enzymes containing the heme group (Singh et al., 2006; Dua et al., 2010; Nath et al., 2011). Similar to the previous studies (Rajesh et al., 2003; Asghari et al., 2017; Armandeh et al., 2021), our findings showed that phosphine significantly impaired the complexes I and IV activity after 12 and 24 h, whereas complex II activity was not significantly reduced. Compared to the corresponding AIP group, EDA treatment at doses of 30 and 45 mg/kg improved complex I and IV activity in 24 and 12 h. Interestingly, treatment with EDA at 45 mg/kg increased boosted complex IV activity to near that of the control group after 24 h. These findings support previous research that found that EDA effectively reduces mitochondrial swelling, preserves acceptable myocardial ATP content, and decreases cytochrome-c release (Rajesh et al., 2003). It is also shown that EDA protects lipid peroxidation and mitochondrial swelling from ischemia/reperfusion-induced mitochondrial dysfunction (Watanabe et al., 1994; Crompton, 1999). This indicates that the EDA’s primary pharmacological target is most likely mitochondria.

According to several investigations, apoptosis and necrosis are induced by phosphine in cardiac cells (Anand et al., 2011; Jafari et al., 2015; Haghi-Aminjan et al., 2018). Possible mechanisms of phosphine-induced apoptosis include an increase in cytochrome C release, a decrease in mitochondrial membrane potential (MMP), and an increase in mitochondrial permeability transition pore (MPTP) opening (Shah et al., 2009; Anand et al., 2011). In the current study, flow cytometry analysis found that AlP poisoning significantly decreased the live cells and raised apoptotic cells in the heart tissue cells. EDA treatment enhanced the percentage of viable cells in all doses.

Interestingly, no significant difference was observed between the 45 mg/kg EDA dose and the control group in the rate of viable cells. This impact could be due to its potent free radical scavenging capabilities, or it could be due to the prevention of mitochondrial permeability transition pore opening and cytochrome C release (Watanabe et al., 1994; Crompton, 1999). In line with the findings of our study, it is shown that EDA can have protective effects against apoptosis and inflammation caused by Ca^+2^ overloads, ROS production, and iNOS expression in cardiovascular disease (Kikuchi et al., 2013b).

The previous study has shown that phosphine can significantly change oxidative stress biomarkers. It has been demonstrated that phosphine increases ROS and lipid peroxidase levels by disrupting the electron transfer chain. This increases free radical generation and changes in antioxidant mechanisms (Kariman et al., 2012; Anand et al., 2013). Various studies have shown that EDA has potent antioxidant effects, reducing hydroxyl radicals, superoxide radicals, and hydroxyl radical-dependent lipid peroxidation (Watanabe et al., 1994; Yamamoto et al., 1997). Our findings showed that phosphine significantly increased the ROS level, and treatment with EDA as a potent radical scavenger significantly decreased the ROS level in the heart tissue at all doses (20, 30, and 45 mg/kg).

Ventricular dysfunction deteriorates and myocardial necrosis occurs as a result of acute myocardial infarction brought on by reduced coronary artery flow (Feng et al., 2012). For years, enzymes like LDH and troponins have served as markers for the diagnosis of acute myocardial infarction (Aydin et al., 2019; Danese and Montagnana, 2016; Mythili and Malathi, 2015). Lactate dehydrogenase (LDH) converts pyruvate to lactate in anaerobic situations such as hypoxia or aerobic pathway abnormalities. Lactic acidosis occurs when the body’s lactate level rises due to a change in the aerobic metabolism to anaerobic (Rogatzki et al., 2015). In line with the previous studies, phosphine poisoning significantly increased cardiac tissue lactate levels. In the previous literature, it has been shown that EDA reduces cardiac biomarkers with potent antioxidant effects (Hassan et al., 2016). Our findings show that treatment with EDA considerably reduces the lactate level produced in phosphine-poisoned rats. This effect on declining the lactate level may be due to improved heart function (LVEF, SV, CO) and preservation of mitochondrial function by EDA during AlP poisoning, which plays a crucial role in elevating tissue perfusion (Hassan et al., 2015; Hassan et al., 2016).

The cTnI is a specific and highly sensitive biomarker for myocardial cell injury and myocardial infarction (Acikel et al., 2003). Studies have shown an association between phosphine intoxication, elevated cTnI levels, and histological changes in damaged myocardial tissue (Asghari et al., 2017; Armandeh et al., 2021). In the present study, the heart tissue cTnI level was significantly enhanced by phosphine poisoning compared to the control group at both times (12 and 24 h), indicating degenerative changes in the myocardial cell membrane. After 12 and 24 h, EDA at all dosages (20, 30, and 45 mg/kg) significantly reduced this biomarker’s level, indicating a decline in myocardial damage.

Patients exposed to phosphine, intentionally or accidentally, die within a short time because there is no specific antidote for this toxicity, and there is not enough time for specialists to provide supportive care. This study showed that EDA, which has high antioxidant, anti-apoptotic, and anti-inflammatory actions on cardiac cells, can attenuate the toxic effects of AlP and increase survival time in poisoned rats.

The main limitation of the current study was that, due to the high mortality rate of severe AlP poisoning, it was impossible to study at doses higher than LD_50_. The strengths of this study were the use of appropriate analytical methods and the analysis of biochemical and molecular experiments conducted at 12 and 24 h following poisoning, which have not been performed in analogous studies with EDA.

## 5 Conclusion

According to the findings of this study, AlP-induced hemodynamic, electrocardiographic, echocardiographic, molecular, and biochemical abnormalities in the heart were improved by EDA administration. Also, the mean survival time was increased following EDA treatment, which can be attributed to the EDA’s protective effects against diverse underlying mechanisms of phosphine-induced cardiac toxicity. This study shows that EDA, with its advantageous features, can play a potential role in reducing cardiac manifestations caused by phosphine poisoning. Therefore, as an adjunct choice, EDA is a good candidate for treating AlP poisoning and other supportive treatments. Despite possible efforts to find an antidote for phosphine poisoning, no results have yet been achieved. Therefore, considering that the main mechanism of phosphine toxicity is severe oxidative stress and disturbance of the electron transport chain, a drug such as EDA, a powerful scavenger, can reduce the severe toxicity caused by phosphine. Although intraperitoneal EDA administration effectively treats AlP poisoning, EDA IV administration is faster and more efficacious. Therefore, a similar investigation with EDA IV administration is required. Also, due to the effect of drug monotherapy on this toxicity, it is recommended to evaluate the treatment with edaravone and typical managements like catecholamines and other antioxidant drugs for a synergistic effect. It is noteworthy that further research utilizing various clinical trials and animal models is needed to examine the mechanism of EDA capability for managing phosphine poisoning.

## Data Availability

The original contributions presented in the study are included in the article/Supplementary Material, further inquiries can be directed to the corresponding author.
